# Addressing Climate‐Related Mental Health Challenges Among Children, Youth, and People Living With Disabilities: A Population Mental Health Commentary

**DOI:** 10.1029/2025GH001597

**Published:** 2026-03-11

**Authors:** Azar M. Abadi, Daniel Dodgen, Susan Clayton, Rhonda Moore, Kimberly Boller, Dennis P. Stolle

**Affiliations:** ^1^ Environmental Health Sciences Department School of Public Health, University of Alabama at Birmingham Birmingham AL USA; ^2^ Washington DC USA; ^3^ Psychology Department The College of Wooster Wooster OH USA; ^4^ Bethesda MD USA; ^5^ American Psychological Association Washington DC USA

**Keywords:** population mental health, disabilities, climate change, psychological resilience, vulnerable population, resilience strategies

## Abstract

Climate change threatens mental health through acute and chronic stressors. Vulnerable groups—especially children and people with disabilities—face increased risks due to developmental and structural inequities. Most mental health systems, built for individual care, lack capacity to address climate‐related distress. This commentary outlines key mental health pathways and calls for a population‐based approach. We highlight climate anxiety among youth and gaps in including neurodiverse populations in research and planning. Culturally grounded strategies—such as school‐based support, community engagement, and integrating lived experience into policy—build resilience. We call for collaboration and evaluation of interventions aimed at addressing climate distress through community‐driven systems.

## Plain Language Summary

Climate change is harming people's mental health; children and those with disabilities are particularly vulnerable. Extreme weather and worry about the future can lead to anxiety, stress, and other issues. Current mental health systems are not built to support large number of people facing group‐level threats such as the climate crises. This paper calls for a new approach that focuses on whole communities, not just individuals. We suggest ideas such as school‐based programs, peer support, and better planning for people with disabilities, including those who are neurodiverse as well as the research needed to guide this work. We also highlight the importance of local involvement, early support, and using technology to improve access. Everyone should be included in the response to climate change.

## Key Points

- Climate‐related psychological distress is a growing population mental health risk for children and people with disabilities
- Near‐term action is feasible by integrating population mental health into existing climate adaptation, education, and emergency systems
- Long‐term resilience requires sustained cross‐sector investment in inclusive, climate‐informed mental health systems

## 1 Introduction

More than half the U.S. population considers climate change a significant source of stress (American Psychological Association, 2024). In a cross‐national survey, 59% of children and young people around the world said they were very or extremely worried about climate change, and 84% were at least moderately worried (Hickman et al., 2021). In a U.S. survey, 85% of young people in the U.S. were at least moderately worried and 43% said their worries affected their mental health (Lewandowski et al., 2024). Over 45% worldwide, and 38% in the U.S., report that their feelings about climate change negatively affect their daily life and functioning (Hickman et al., 2021; Lewandowski et al., 2024). Many groups, including persons living with disabilities and children, are at heightened risk of psychological distress from climate change (Stein et al., 2024).

The number of people experiencing climate‐related psychological distress is likely to grow as the planet warms and severe weather events become more common. Current mental health systems, which primarily focus on individual‐level care, may not have the capacity to cope. New and creative systems for addressing climate‐related psychological distress at a population level will become increasingly important. This approach will require scaling up evidence‐based interventions, while considering lessons from implementation science to maintain their effectiveness (Dodge et al., 2024).

**Figure 1:**
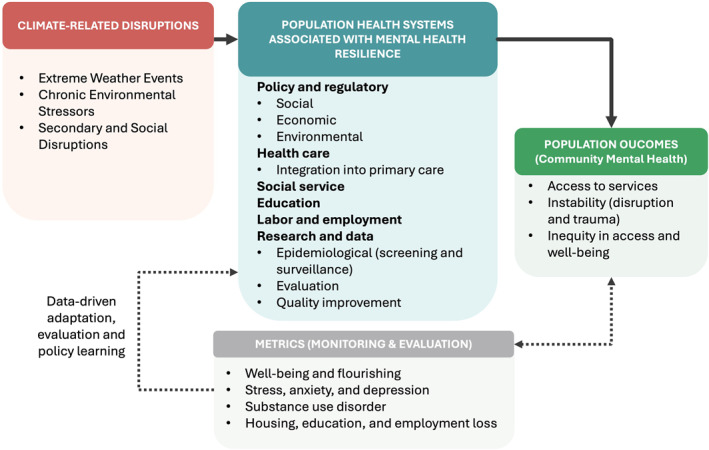
Pathways from population‐level systems and climate disruptions to address health equity and community mental health. The figure illustrates the multiple systems associated with mental health resilience and documented population‐level metrics aligned with climate‐related disruptions. These associations can be mitigated by system‐level interventions and collaboration. Examples include provision of stability through cross‐sector coordination, policy and associated flexibility in funding and regulatory requirements, integration of mental health supports into primary and community‐based care, and adaptation policies.

To conceptualize such an approach, population health provides a useful framework for understanding how systems can influence mental well‐being at scale. Population health refers to “the health outcomes of a group of individuals, including the distribution of such outcomes within the group” (Kindig & Stoddart, 2003). It emphasizes that health is not solely the result of individual actions or clinical care, but is created through cultural, economic, environmental, and social conditions. The systems and pathways associated with population health and resilience are cross‐sector and complex, implicating a range of outcomes at the community level, including population mental health (National Academies of Sciences, Engineering and Medicine, 2024). Health equity is a central population health outcome, and it is also critical to include in climate‐related metrics. Multiple population‐level systems are associated with key population outcomes through cross‐sector pathways (Figure 1). Systems include policy and regulatory, health care, social service, education, and labor and employment. Population‐level research and data contribute to management and evaluation of health system functions and health equity.

Drawing from existing population health and prevention frameworks that address physical health, a population mental health approach moves beyond individual conditions and treatments to a focus on the distribution of mental health across entire populations (defined variously by geography, culture/ethnicity, gender, and ability; Dodge et al., 2024). A population mental health approach includes an emphasis on prevention and health promotion (National Academies of Sciences, Engineering, and Medicine, 2025) that addresses upstream social and structural determinants of health and systems with a history of perpetuating health disparities (e.g., Solar & Irwin, 2010).

Applying a population mental health framework to climate‐related disruptions requires mapping the pathways among those disruptions, features and functioning of systems and sectors, and population‐level measures. At the same time, a population mental health framework does not exclude treatment for severe mental health conditions that may arise from climate‐related disruptions or the compounding of climate anxiety with other factors that affect mental health. As depicted in Figure 1, climate‐related disruptions are associated with mental health outcomes that are identifiable using population‐wide metrics of community mental health. Despite progress in expanding the population mental health measurement toolbox to include measures of the prevalence of climate anxiety and related outcomes at different population levels, more needs to be done to develop and include strengths‐based metrics in addition to those that reflect deficits and decreases in population mental health.

Addressing climate‐related psychological distress globally will require culturally informed, cost‐effective, and widely accessible interventions. This requires assessing and adapting the population‐level systems, supports, and respective outcomes and metrics to track innovations focused on provision of community‐wide interventions. For example, if the business sector collaborates with the government and communities to address challenges in digital access to community needs assessments before a climate event occurs, deployment and use of community‐wide interventions and strategies to address needs of under‐resourced areas could reduce negative impacts of climate on population mental health. Another example focuses on creative solutions for supporting the psychological well‐being of youth around climate change issues. These may include technology (online support, text‐based support, gamification), peer support programs, school‐based support, and alternative service providers) (Dodge et al., 2024).

Throughout this paper, we use the term mental health to refer specifically to emotional, psychological, and social well‐being, including conditions such as depression, anxiety, and trauma‐related disorders. We acknowledge that behavioral health is a broader term often used in public health and clinical settings to encompass both mental health and substance use disorders, as well as health‐related behaviors (American Medical Association, 2024). However, to increase clarity and focus we address mental health outcomes in the context of climate change and do not include substance use or other behavioral health domains.

In the following pages, we present a narrative, conceptual commentary on the mental health impacts of climate change, with particular attention to children and people living with disabilities who experience disproportionate vulnerability. Drawing on the authors' interdisciplinary expertise and selected examples from recent research and policy‐relevant reports, this article aims to: (a) highlight current evidence on how climate change affects mental health and psychological well‐being in these populations; (b) apply a population mental health framework to interpret these impacts within broader social, environmental, and systems‐level contexts; and (c) outline implications for policy, practice, and future research that support population‐level mental health responses. The intent is not to provide a comprehensive or systematic review, but to present salient evidence and perspectives and to clarify climate‐related psychological distress as a population‐level threat—rather than solely an individual clinical concern—in order to inform scalable, preventive, and systems‐oriented approaches to mental health adaptation in a changing climate. The commentary elaborates on how this approach is particularly suited for addressing the needs of children and people with disabilities.

## 2 Mental Health Impacts of Climate Change

Climate change threatens mental health through multiple channels. Extreme weather events associated with climate change are clearly associated with increased levels of Post‐Traumatic Stress Disorder (PTSD), anxiety, depression, substance abuse, sleep problems, and domestic and interpersonal violence (Clayton & Crandon, 2025). These effects may linger for months or years after the event (Goldmann & Galea, 2014).

Slower, chronic changes in climate such as higher temperatures, changing patterns of precipitation, and decreased air quality also threaten mental health. Recent large‐scale and longitudinal studies show that sustained exposure to extreme heat and degraded air quality contributes to elevated risks of anxiety, depression, suicide, and psychiatric hospitalizations (Baecker et al., 2025; Jung et al., 2025; Mitchell et al., 2024). For example, in a cross‐sectional study of 86,609 emergency‐department visits during the 2020 California wildfires, each 10 μg/m^3^ increase in wildfire‐specific PM_2.5_ was associated with an 8% increase in visits for mental‐health conditions (Jung et al., 2025), while a global systematic review found a 2.2% rise in mental‐health‐related mortality per 1°C increase in ambient temperature (Baecker et al., 2025). Longitudinal cohort evidence from Australia from longitudinal research further indicates that repeated exposure to climate disasters is associated with cumulative declines in population‐level mental‐health scores (Mitchell et al., 2024). Taking a broad perspective on mental health, there is also evidence that cognitive and social functioning are threatened (Miles‐Novelo & Anderson, 2022; R. J. Park et al., 2020).

Some of the most widespread impacts of climate change are indirect. Climate change can have economic impacts, for example, if property is damaged or loses value due to rising sea levels or wildfires, or if occupations and economic activity are threatened. Involuntary displacement is a consequence for many people who live in geographically vulnerable areas. Food insecurity is on the rise due to climate change (Gaskin et al., 2017; Lindsay et al., 2023; Stein et al., 2024; UNDRR, 2023). Each of these processes is associated with worse mental health, and often with disruption of the social ties that can help people to be resilient (Clayton & Crandon, 2025).

National and international surveys, as mentioned above, show high levels of concern and worry about climate change and its potential to have negative impacts (e.g., Leiserowitz et al., 2024). Although such concerns are grounded in lived experience and may promote effective action to mitigate and adapt to climate change, a high level of concern can threaten mental health (Clayton, 2020). Climate anxiety has been found to be associated with clinical levels of anxiety, depression, and other mental health problems in countries around the world (Gago et al., 2024; Ogunbode et al., 2022).

Other impacts include threats to place identity and stresses on social relationships (Clayton, 2024). The profound inequities in the distribution of climate change impacts constitute their own threat to mental health. Factors like economic inequalities, social inequalities, physical limitations, and geographic vulnerability all affect the extent to which people experience the impacts of climate change as well as their ability to be resilient.

**Figure 2:**
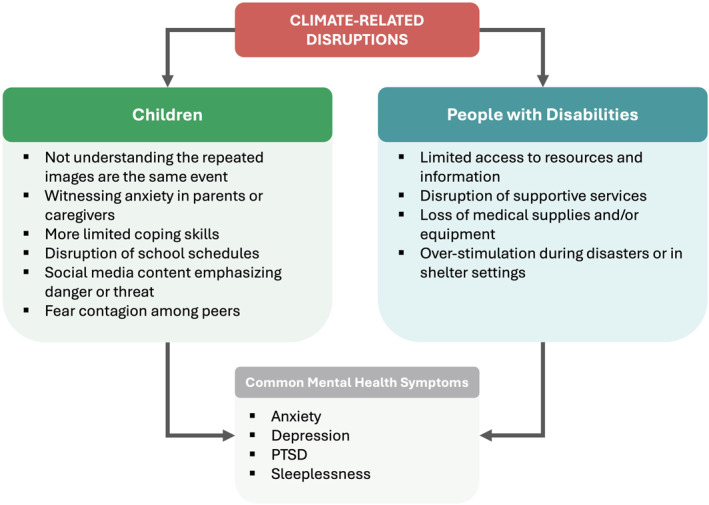
Differing pathways linking climate‐related disruptions to mental health symptoms among children and people with disabilities.

Two vulnerable groups, children and people with disabilities, experiences of climate‐related disruptions are different but often lead to similar mental health symptoms, including anxiety, depression, PTSD, and sleeplessness (Figure 2). Next, we highlight specific findings about each group separately followed by how a population health perspective broadens the types of approaches communities can take to adaptation (such as disaster preparedness) and foster resilience.

## 3 Children as a Disproportionately Affected Population

### 3.1 Developmental Sensitivity and Risk Amplification

As Clayton and her colleagues (Clayton et al., 2023) summarized: “The impacts of climate change intersect with and compound other factors that threaten youth mental health, including child development, parental health, increasing rates of depression and suicide, racism, poverty, housing security, adequate nutrition, and access to medical care, as well as major societal issues like COVID‐19, gun violence, social media, and much more.”

Age and developmental stage are particularly important because they influence how children process, interpret, and react to information. For example, if young children see repeated replays of a wildfire on the news, they may not understand that it is a single event rather than multiple events. Furthermore, they may not know to turn off the television or disengage from social media when it makes them anxious. Children and adolescents may have not yet developed a full range of coping skills, thus increasing their risk for anxiety, depression, bipolar disorder, cognitive function impairment, interpersonal aggression, and other mental health impacts (Clayton et al., 2023; Figure 1).

### 3.2 Climate‐Anxiety, Information Exposure, and Cumulative Stressors

Adolescents have unique risks due to “climate‐anxiety” and other factors. This climate anxiety is related to exposure through social media as well as actual exposure to weather‐related disasters. Auchincloss et al. (2024) in a U.S.‐based cross‐sectional study, found that adolescents exposed to 45‐plus days of weather‐related disasters in a school year were at a 15% greater risk of developing mental health problems than those exposed to fewer days (Auchincloss et al., 2024). As described above, a global study of about 10,000 young people aged 16–25 across 10 countries found that nearly 40% reported that their concerns about climate change made them hesitant or unwilling to have children (Hickman et al., 2021). A review by Lawrance et al. (2022) summarized global evidence demonstrating that a large proportion of survey respondents report strong emotional distress related to the climate crisis. Consistently, studies showed that more than 60% of respondents reported feeling afraid, sad and/or anxious, while at least 50% reported feeling angry, powerless, helpless and/or guilty about the climate (Lawrance et al., 2022).

### 3.3 Structural Inequities and Intergenerational Burden

This impact is different for different groups. The National Climate Assessment found that systemic racism and discrimination exacerbate climate impacts on human health (USGCRP, 2023). This cuts across Black, Indigenous, and People of Color (BIPOC) communities, particularly Indigenous Peoples, as well as sexual and gender minorities. Consequently, children who are also members of historically marginalized groups may experience a greater cumulative burden than they would otherwise experience (Clayton et al., 2023). Climate change also creates intergenerational inequities since younger generations are experiencing more extreme weather events than older ones.

## 4 People Living With Disabilities as a Disproportionately Affected Populations

### 4.1 Differential Exposure and Service Disruption

Persons with disabilities (PWDs) constitute about 16% of the world's population, with 80% of PWDs living in the Global South. They are also the most frequently affected by natural hazards, climate‐induced disasters, and global health emergencies (Gaskin et al., 2017; Lindsay et al., 2023; Stein et al., 2024; UNDRR, 2023). However, while most of the literature on this topic has tended to emphasize physical disabilities, considerably less is known about invisible disabilities including neurodivergent conditions such as autism spectrum disorder (ASD), attention deficit hyperactivity disorder (ADHD), and how they interact with the climate crisis (Watfern & Carnemolla, 2024).

### 4.2 Research Gaps in Understanding Neurodivergent and Invisible Disabilities

There are significant gaps in this literature with much of what we know coming from the field of natural disasters, which also seldom discusses the differential impacts of climate change on intersectional neurodivergent conditions and experiences. Stein et al. (2024) have argued that climate change creates additional comorbidities that are unique to PWDs and exacerbates existing harms, both within countries and across regions, unequally affecting low‐income settings (Stein et al., 2024). Additional research is needed that explores how climate change creates additional intersectional disabilities and exacerbates existing harms across diverse cultural climate change contexts. Other research should use a disability human rights approach that actively incorporates the lived perspectives and experiences of people with disabilities to develop climate change solutions. Research is also crucially needed on developing the climate resilience of front‐line disability communities (Stein et al., 2024).

### 4.3 Health and Functional Challenges During Climate Extremes

This gap in the literature is becoming increasingly important, as diagnoses for neurodivergent conditions such as ASD and ADHD have been on the rise, particularly in countries with improved surveillance methods and diagnostic criteria (Nori‐Sarma & Galea, 2024; Park et al., 2024). Research shows that people with intellectual disability and other mental health disorders are also at greater risk and should be included in groups considered at high risk for heat exposure (Park et al., 2024). This has implications for psychiatric medicine intake since common drugs for anxiety, depression and other mental health disorders can cause problems with thermoregulation and may impair cognitive functioning in extreme heat (Dodgen et al., 2016). One of the more glaring gaps in the disaster preparedness and response research is the failure to account for the sensory and executive functioning challenges of neurodiverse populations in the context of disasters (Hamstead, 2024).

### 4.4 Lived Experience, Inclusion, and Data Needs

Regrettably, the lived experiences of neurodivergent individuals have also not been historically captured as part of inclusive planning, accessible information, early warning systems, and transportation in and across disaster settings (Stein & Stein, 2022; Stein et al., 2024). Understanding and including the lived experiences of neurodiverse populations is essential for informing collaboration and inclusive climate policies and practices. Lived experience research is also becoming the norm across mental health research and should be reflected in climate and mental health research, policy and practice (Gilbert et al., 2025; Muchamore et al., 2024).

## 5 Building Resilience in Disproportionately Affected Populations

Approaches to supporting mental health and well‐being amid climate‐related challenges must consider the disproportionate impact on vulnerable populations, including children, youth, and people with disabilities (Gamble et al., 2016). The bioecological framework for human development offers a foundation for identifying and testing evidence‐based and promising strategies across individual, family, school, workplace, and community levels (Bronfenbrenner & Morris, 2006). Solutions should aim to build resilience and promote thriving within these interconnected systems, shaped by policy and programmatic decisions. Strong relationships, social engagement, and the involvement of health and allied professionals have emerged as key supports for resilience, particularly in the face of extreme weather events and ongoing climate stressors.

### 5.1 Children and Youth

Recent efforts by developmental scientists and policymakers emphasize strengthening early care and education systems to support children, youth, and caregivers before, during, and after extreme weather events (Ponguta et al., 2023). These systems, already grounded in evidence‐based practices, offer a foundation for climate resilience without requiring entirely new infrastructure. Aligning early childhood systems (ages 0–8) with the United Nations Sustainable Development Goals (sdgs.un.org/goals) enables integrated responses to climate‐related disruptions and leverages work already in progress across the globe.

Local policies that maintain education and nurturing adult‐child interactions during disasters must also address the mental health of parents and educators. Adapted resources—originally developed for emergencies like displacement caused by armed conflict—can support families facing climate‐related instability. For example, age‐appropriate emergency kits have been used globally to promote resilience and social‐emotional learning through games and educational materials.

However, support remains inconsistent. Analyses show that children and youth are often overlooked in climate policies (Balel O., 2025; Turay B., 2025). A broader call to action urges health professionals to advocate for equity‐focused climate responses (Epel et al., 2025). Initiatives like the American Public Health Association's “Climate and Health Youth Education Toolkit” equip professionals to educate youth and promote policy advocacy. While promising, these efforts require further research to assess their effectiveness and scalability.

### 5.2 People Living With Disabilities

Although frameworks exist to guide support for people with disabilities, evidence on building psychological resilience remains limited. Gamble et al. (2016) identify three critical factors—exposure, sensitivity, and adaptive capacity—that shape health vulnerabilities. These are especially relevant for people with disabilities, who often depend on others for communication and emergency response. Social networks, including family, faith communities, and workplaces, have been shown to enhance resilience (Fox et al., 2010).

Yet, global climate policy remains largely exclusionary. Fewer than 40% of countries mention people with disabilities in their climate adaptation plans, and most references lack meaningful inclusion (Jodoin et al., 2025). Calls for disability justice emphasize recognizing the diversity within disability communities and addressing intersecting social determinants like poverty and racial discrimination (Engelman et al., 2022). Despite well‐documented barriers—such as inadequate communication and evacuation planning—few solutions have been rigorously tested or scaled. Further research and advocacy evaluation are needed to inform effective, inclusive climate responses (Stein et al., 2024).

## 6 Policy and Practice Implications for Collective Well‐Being

The policy and practice implications of a population mental health approach can be organized by time horizon and along a continuum of levels of action. This approach needs to address both community‐wide needs and the needs of specific groups such as children and people with disabilities. In the near term, opportunities exist to integrate mental health considerations into existing climate adaptation, education, disability, and emergency response systems. Over the longer term, structural investments are needed to build accessible, responsive mental health infrastructure that is resilient to climate disruption. Along this continuum, opportunities range from actions at the level of individuals and families to potential national or international policy change. Across all levels, however, effective responses depend on cross‐sector collaboration among environmental, health, education, and social service systems.

Overall, to effectively cope with the mental health burdens stemming from climate change, there is a need for policies that focus on both collective psychological well‐being and individual‐level care. Specifically, we need to develop systems of care that reach the full population at regular intervals across the lifespan to identify mental health risks and needs so that such risks and needs can be addressed early, before they reach the stage of a diagnosable mental illness (Dodge et al., 2024). Building and maintaining systems of care will necessitate cooperation and support among schools, communities, and neighborhoods.

While these strategies are a critical first step, it is unlikely that a one‐size‐fits‐all solution will support collective psychological well‐being in the face of climate change, particularly for children and people with disabilities. Local community involvement in the development and implementation of programs is critical to ensure that cultural norms and values are respected in program administration and that the needs of real people in that community are addressed. For example, communities with high percentages of seniors will also have higher rates of disability, even though seniors may not self‐identify as having disabilities. Community‐based approaches cannot be solely deficit based. Understanding existing strengths and resources within communities, leveraging them, and building upon them is critical. In all these efforts, we must emphasize access to services and utilize innovations such as telehealth to promote greater access.

Many communities have already invested in programs to address related concerns. School‐based mental health programs, which have positive outcomes (Richter et al., 2022), exist in many schools. Emergency management programs have promoted community resilience, inclusive planning, and improved data‐sharing protocols (National Institute of Environmental Health Sciences, 2025; Slemp et al., 2020). These existing programs can be leveraged to create a “system of systems” to promote psychological well‐being in the face of climate change. However, there is a long way to go in linking interventions to meaningful population mental health outcomes and determining their cost‐effectiveness. To do so requires the use of appropriate community‐wide data and collection of the true costs of such interventions.

Further, such a system of care should not be implemented without rigorous program evaluation and ongoing quality improvement. These evaluations should include objective measures of psychological well‐being at both the individual and population levels, as well as assessments of the subjective, lived experiences of all participants in the system. This includes both those receiving care and those involved in delivering, managing, or overseeing the services.

Continuing research is necessary to inform the development of policies and programs. Gaps in the research base must be identified and addressed, including, for example, a lack of data on neurodiverse populations. While such research is challenging, it is essential to ensure that entire populations are served, with no one left behind.

Despite rapid growth in climate–mental health research, several research gaps limit translation of existing evidence into scalable and equitable population approaches. These include: (a) limited data on neurodivergent populations and other “invisible” disabilities across diverse contexts; (b) insufficient evaluation of community‐wide interventions using population mental health metrics, including strengths‐based indicators; (c) limited evidence on cost‐effectiveness and implementation at scale across real‐world systems (e.g., schools, social services, emergency management); and (d) underrepresentation of lived experience and participatory approaches in the design of climate‐resilient mental health supports. Addressing these gaps is essential for building a “system of systems” that can be evaluated, improved, and sustained over time.

## 7 Conclusions and Implications

This commentary highlights evidence and perspectives to advance a population mental health framing of climate‐related psychological distress, rather than to comprehensively review or systematically evaluate the literature. As reflected throughout the manuscript, available evidence remains uneven across populations and contexts, with particularly important gaps for people living with disabilities—including neurodivergent populations—and for low‐resource and underrepresented regions. In addition, while the recommendations proposed here are grounded in established public health concepts such as prevention, resilience, and cross‐sector systems of care, many have not yet been evaluated as climate‐specific population mental health interventions. These limitations highlight the need for more inclusive data, participatory research that centers lived experience, and rigorous evaluation of population‐level strategies designed to support mental health in a changing climate.

Despite these limitations, the implications are clear. Climate change poses growing mental health risks, especially for children and people with disabilities. Systems focused on individual care are insufficient to meet the scale of need. A population mental health approach—grounded in health equity, early intervention, and community resilience—is urgently needed. This includes school‐based programs, inclusive planning for neurodiverse individuals, and broader access through technology. Research gaps, especially regarding vulnerable populations, must be addressed. We call for interdisciplinary collaboration to design, evaluate, and scale solutions. Building climate‐resilient mental health systems is essential to protect well‐being in an increasingly unstable climate.

## Conflict of Interest

The authors declare no conflicts of interest relevant to this study.

## Data Availability

Data were not used, nor created for this research.
